# Trastuzumab deruxtecan in HER2-positive metastatic breast cancer with severe hepatic dysfunction: a case report and narrative literature review

**DOI:** 10.3389/fonc.2026.1753463

**Published:** 2026-09-14

**Authors:** Giuseppe Saltalamacchia, Riccardo Gerosa, Jacopo Canzian, Chiara Benvenuti, Caterina Giannitto, Flavia Jacobs, Mariangela Gaudio, Benedetta Tinterri, Chiara Miggiano, Giovanna Masci, Rita De Sanctis, Rosalba Torrisi, Armando Santoro

**Affiliations:** 1 Humanitas Cancer Center, IRCCS Humanitas Research Hospital, Milan, Italy; 2 Department of Biomedical Sciences, Humanitas University, Milan, Italy

**Keywords:** HER2-positive breast cancer, liver dysfunction, metastatic breast cancer, trastuzumab deruxtecan (T-DXd), visceral crisis

## Abstract

Trastuzumab deruxtecan (T-DXd) has transformed the management of Human Epidermal Growth Factor Receptor 2 (HER2)-positive metastatic breast cancer (mBC). However, its role in visceral crisis (VC) remains unclear due to exclusion from clinical trials and limited real-world evidence. We report the case of a 40-year-old woman with HER2-positive mBC who after first-line therapy developed severe hepatic dysfunction due to liver progression, consistent with hepatic VC. Following multidisciplinary discussion and informed consent, T-DXd as second line therapy was initiated at a reduced dose (3.2 mg/kg), leading to rapid normalization of liver function, symptom relief, and radiologic response. Subsequent stepwise escalation to the standard dose (5.4 mg/kg) was well tolerated, with durable disease control and no additional toxicity. Our findings, in this otherwise underexplored setting, together with similar experiences reported in the literature, support T-DXd as a safe and effective therapeutic option for HER2-positive mBC complicated by hepatic VC. Further evidence is needed to better inform clinical practice in terms of optimal, tailored dose-escalation strategies in this context.

## 1 Introduction

Breast cancer (BC) is the most common malignancy in women and the leading cause of cancer-related mortality (1). Most BC patients are diagnosed at early stage (eBC), where multimodal therapy offers a realistic chance of definitive cure (2). Conversely, in about 10-15% of patients with diagnosis of metastatic breast cancer (mBC), disease onset, or progression present with a life-threatening condition, known as visceral crisis (VC) (3, 4). The 6th and 7th International consensus guidelines for the management of advanced breast cancer (ABC 6 and 7) define VC as severe organ dysfunction assessed through clinical signs, symptoms, and laboratory findings, requiring rapid disease control. (4). The same guidelines provide organ-specific definitions of VC; for instance, liver VC is characterized by a rapid increase in bilirubin >1.5× the upper limit of normal without biliary obstruction. Notably, a more subjective and less well-defined scenario, termed ‘impending visceral crisis’, describes cases where formal VC criteria are not yet fulfilled but, without timely and effective intervention, is foreseen to happen. (5). Given the lack of standardized definition and usually representing an exclusion criterion from clinical trials, evidence to guide VC optimal management remains extremely limited, particularly in aggressive biological subtypes such as HER2-positive disease (6). Historically, VC treatment has relied on single-agent chemotherapy or, in selected cases, combination chemotherapy with or without targeted agents (e.g., anti-HER2 or anti-VEGF therapies), but no regimen has emerged as clearly preferred or most effective (4). In recent years, novel emerging agents such as antibody–drug conjugates (ADCs) are revolutionizing mBC management by providing more effective treatment options. Among them, the cutting-edge anti-HER2 ADC, trastuzumab deruxtecan (T-DXd), has indeed shown significant survival benefits and higher responses compared with standard regimens in HER2-positive mBC (7, 8), findings further supported by real-world data (9–12). Importantly, it has demonstrated robust efficacy even in challenging context such as symptomatic, active, or untreated brain metastases and leptomeningeal disease in both HER2-positive and HER2-low tumors (13–16). Nonetheless, despite representing a possible ‘game changer’ in VC scenarios, the role of T-DXd in controlling extracranial life-threatening conditions, remains undertested. Indeed, from a pharmacokinetic standpoint, T-DXd is primarily metabolized by hepatic cytochrome P450 (CYP450), and its labeled safety profile includes transaminase and bilirubin elevations, with any-grade hepatic events reported in approximately 25–35% of patients (10, 11, 17). However, pivotal trials evaluating T-DXd have generally excluded patients with severe baseline hepatic dysfunction or overt VC, leaving a critical evidence gap where rapid and effective HER2-directed therapy is most needed. Therefore, we report the case of a young patient with HER2-positive mBC and liver VC treated with second line T-DXd. Additionally, we performed a focused narrative literature review to identify already published and existing evidence. The search was conducted using the PubMed database up to December 2025. We applied the following MeSH and free-text search terms in various combinations: “metastatic cancer”, “Trastuzumab-Deruxtecan”, “hepatic failure”, “liver dysfunction”, “visceral crisis”, “rapidly progressing disease”, “symptomatic visceral metastases” and “symptomatic non-visceral metastases”. Only articles published in English and reporting clinical data (case reports, case series, observational studies, clinical trials) were considered. Titles and abstracts were screened for relevance, and full-text review was performed for all selected studies. References from included articles were also examined to identify additional eligible reports. By expanding and contextualizing the available literature together with our case report, we aim to offer meaningful clinical insights into a largely unexplored scenario.

## 2 Case description

In May 2020, a 40-year-old woman presented to our institution, IRCCS Istituto Clinico Humanitas Research Hospital (Milan, Italy), with multicentric grade 3 invasive carcinoma of no special type (NST) of the left breast with luminal B-like/HER2-negative profile: estrogen receptor (ER) and progesterone receptor (PgR) positive, HER2 1+ by immunohistochemistry (IHC), and Ki67 30–40%. On July 8, 2020, the patient underwent left mastectomy with ipsilateral lymph node sampling, which confirmed a pathological stage pT3N1(mi) with the same biological profile. The patient subsequently received adjuvant doxorubicin–cyclophosphamide followed by docetaxel, chest-wall radiotherapy (40.5 Gy in 15 fractions), and ovarian function suppression plus exemestane.

The patient remained in regular follow-up until July 2023, when she presented with severe back pain. Spine MRI revealed metastatic lesions at L1 and L2, and CT confirmed widespread disease involving liver, lymph nodes, and other bone sites (sternum, ribs, iliac bones and other). Palliative radiotherapy (30 Gy in 10 fractions) was delivered to the lumbar spine. Biopsy of a parasternal lesion showed a subtype shift to Luminal B-like/HER2-positive disease: ER 90%, PgR <1%, Ki67 15%, HER2 2+ (IHC) with Fluorescence *In Situ* Hybridization (FISH) amplification. In August 2023, she began standard first-line systemic therapy with pertuzumab, trastuzumab, and docetaxel followed by maintenance subcutaneous dual anti-HER2 blockade and endocrine therapy, together with denosumab for bone protection, achieving SD until June 2024.

By July 2024, radiological restaging revealed progression of liver metastases and thoraco-abdominal lymph nodes, and the patient was considered for second-line treatment with T-DXd. A new biopsy was not performed for the need to promptly initiate systemic treatment. At therapy initiation, routine blood tests showed marked hepatic dysfunction with total bilirubin 5.9 milligrams per deciliter [mg/dL] (reference range, 0.3–1.2 mg/dL), Aspartate Aminotransferase (AST) 130 international units per liter [UI/L] (<35 UI/L), Alanine Aminotransferase (ALT) 142 U/L (<35 UI/L), and Gamma-Glutamyl Transferase (GGT) 1373 UI/L (1–37 UI/L). Clinically, her condition was fair, with an Eastern Cooperative Oncology Group (ECOG) performance status of 1. She did not refer other symptoms (e.g. pruritus, fever, abdominal pain), except for grade 1 fatigue. Physical examination revealed no abnormalities, with normal skin appearance, non-icteric sclerae, and no other clinically significant findings. Abdominal ultrasound excluded acute biliary obstruction or other complications. Following multidisciplinary discussion, the medical team recommended T-DXd, with the aim of achieving a rapid tumor response and improving liver function. Given impaired hepatic function, even in the absence of clear guidelines the patients agreed to initiate T-DXd at a reduced dose of 3.2 mg/kg, the lowest approved. She was admitted to the oncology ward for supportive care, including intravenous hydration, oral corticosteroids (prednisone 25 mg once daily with gradual tapering over subsequent weeks) for symptomatic supportive purposes and close laboratory monitoring. Over the following three weeks, liver enzymes and cholestatic markers progressively normalized (as detailed in **Figure 1**) with concurrent improvement in fatigue and general well-being. After three cycles, CT imaging showed an initial dimensional reduction of the hepatic target lesions (**Figure 2**), with stability of all other disease parameters. Given the normalization of liver function and evidence of tumor response, T-DXd was escalated to 4.4 mg/kg for two cycles and subsequently to the full dose of 5.4 mg/kg. By January 2025, after overall seven T-DXd cycles, repeat CT scan showed further reduction of liver target metastases, but also decreased size or disappearance of abdominal-thoracic lymphadenopathies, and remineralization of bone lesions, consistent with a skeletal response. By May 2025, after eleven T-DXd cycles, imaging demonstrated the best treatment response achieved, consistent with a partial response according to Response Evaluation Criteria in Solid Tumors version 1.1 (RECIST 1.1), with target liver lesions decreasing from 33 to 21 mm (segment VII) and from 34 to 18 mm (segment VI), together with further response or resolution (lymphadenopathies) of other disease sites (**Figure 2**) (18). From February to July 2025, the patient continued T-DXd, completing 14 cycles in total, remaining in good clinical condition, with liver function consistently within normal limits. No dose delays or reductions were required during treatment. Serial chest CT scans and routine echocardiography every 6–9 weeks showed no evidence of T-DXd–related interstitial lung disease or cardiac toxicity. No major treatment-related adverse events occurred; the only reported toxicities were grade 2 nausea and G1 mucositis according to the Common Terminology Criteria for Adverse Events, version 5.0 (CTCAE v5.0) (19). In January 2025, suspected osteonecrosis of the jaw occurred during bone-targeted therapy with denosumab, which was therefore discontinued, leading to resolution of the local condition. In August 2025, after a progression-free survival (PFS) of approximately 12 months on T-DXd, radiological restaging documented progression in liver and abdominal lymph nodes. Third-line tucatinib, capecitabine, and trastuzumab was initiated and subsequently discontinued in October 2025 because of hepatic function deterioration without biliary obstruction. The patient was therefore switched to dose-reduced vinorelbine plus trastuzumab but was admitted in early November with sepsis and pancytopenia. Given the progressive clinical decline with poor performance status, the team, together with the patient and caregivers, agreed to transition to exclusive palliative care.

**Figure 1:**
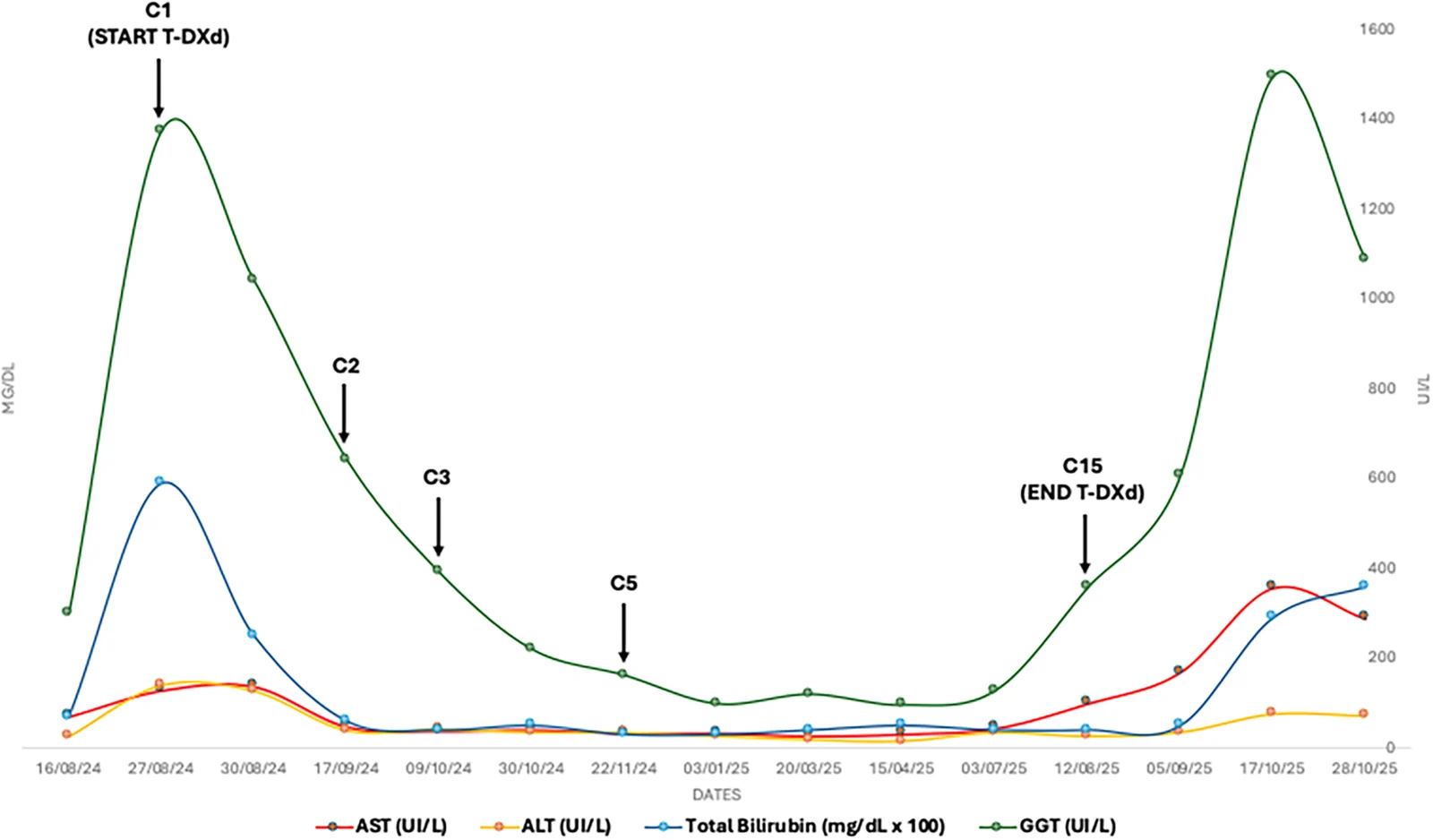
Longitudinal trend of liver function tests after initiation of T-DXd. Liver function parameters (AST, ALT, total bilirubin, and GGT) measured at each time point between August 16, 2024, and October 28, 2025 (last available assessment) are shown. T-DXd was initiated on August 27, 2024 (cycle 1) and the last administration done on August 12, 2025 (cycle 15). The two vertical axes report absolute values according to measurement units: mg/dL for total bilirubin and U/L for transaminases and GGT. Trastuzumab deruxtecan (T-DXd), Cycle Number of T-DXd administration (C), aspartate aminotransferase (AST); alanine aminotransferase (ALT); gamma-glutamyl transferase (GGT), milligrams per deciliter (mg/dL), international units per liter (IU/L).

**Figure 2:**
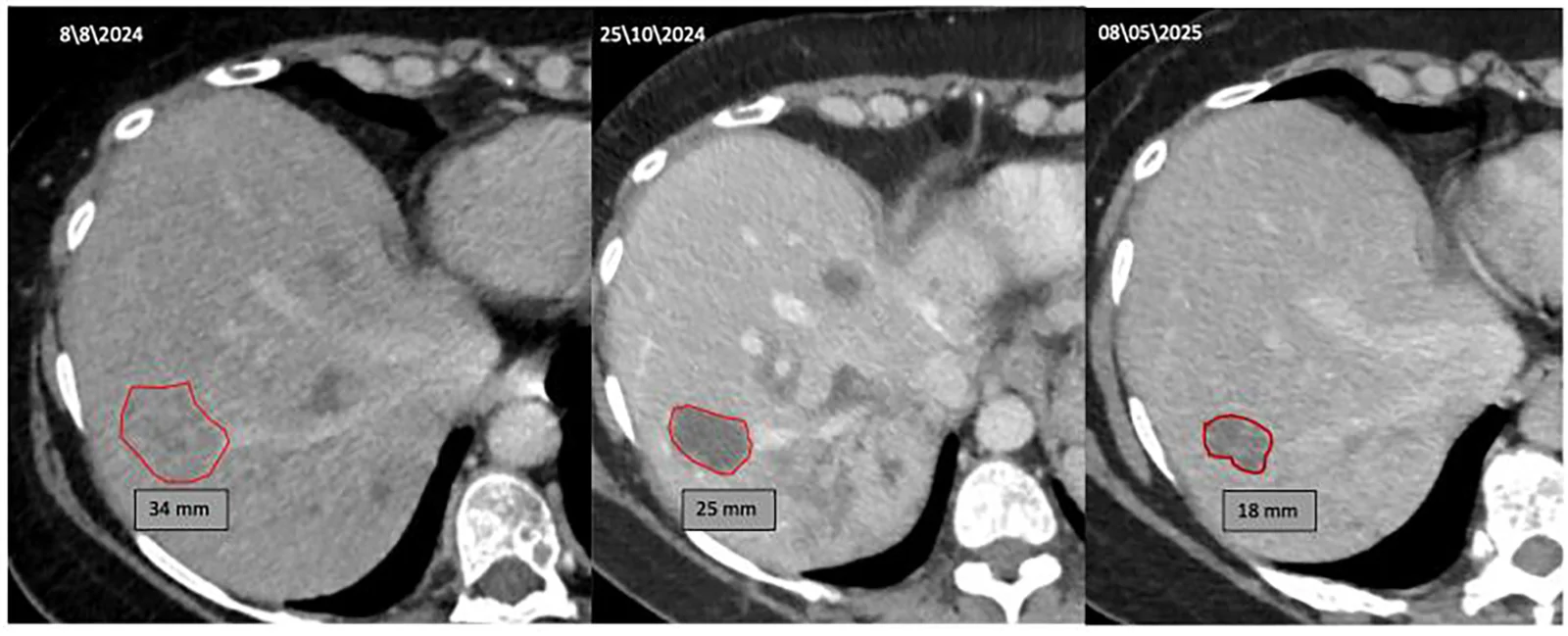
Radiological response of hepatic metastasis to T-DXd therapy. Contrast-enhanced abdominal CT (portal venous phase), axial view. Comparison of scans obtained on August 8, 2024 (left), October 25, 2024 (middle), and May 8, 2025 (right) shows a hepatic lesion in segment VII (outlined in red) with progressive size reduction from 34 mm at baseline (left) to 25 mm at first re-evaluation (middle) and 18 mm at best response (right). The lesion, initially solid, developed central necrosis with a reduced viable component. At first re-evaluation, the adjacent liver parenchyma showed perilesional hypodensity consistent with post-treatment changes, without evidence of new solid tumor infiltration.

## 3 Discussion

In this case report of young woman with aggressive HER2-positive mBC and liver VC, T-DXd provided clear clinical benefit. This included rapid normalization of liver function and cholestasis markers, relief of symptoms, and radiological response. Furthermore, a stepwise dose escalation from 3.2 mg/kg to the full 5.4 mg/kg was well tolerated, with no additional toxicity observed and sustained disease control over 1-year period. In VC setting, rapid and robust disease control is the crucial priority and thus objective response rate (ORR) and time-to-response (TTR) are key endpoints to consider. Both pivotal trials and real-world cohorts report that in pretreated HER2-positive mBC, T-DXd nearly doubles the ORR compared with other anti-HER2 agents and increases complete response (CR) rates, with a very short TTR, often evident at the first scheduled tumor assessment (7, 8, 10, 20). Considering also the first-line setting, T-DXd plus pertuzumab nearly doubled the CR rate versus the “standard of care” docetaxel/trastuzumab/pertuzumab regimen CLEOPATRA regimen (21). Similarly, in pretreated HER2-low and ultra-low mBC, T-DXd achieved better ORR compared with chemotherapy with a comparable TTR (~3 months), although still lacking confirmation from large real-world data (9, 22, 23). Overall, beyond the clear and clinically meaningful survival advantage over standard alternatives across settings and treatment lines, T-DXd’s rapid and remarkable tumor-shrinking efficacy is undisputable. However, the existing literature provides almost no prospective evidence on T-DXd activity specifically in the VC scenario (according to the latest definition), with only a few real-world published cases (summarized in **Table 1**). Specifically, nearly all of those reported on hepatic VC, and none involving other histology in which T-DXd is an approved option (e.g., gastric or lung cancer). Beyond the strict definition of VC, reports of its use in other extracranial settings are lacking, whereas as previously mentioned its intracranial efficacy is well documented (13–16). In terms of efficacy, and consistent with our experience, a rapid clinical response was observed in all cases, with early symptomatic improvement, normalization of liver function abnormalities within the first treatment cycles, and radiologically documented tumor shrinkage. However, our patient’s early clinical and biochemical improvement following the initiation of T-DXd may have been confounded by the administration of oral prednisone during the first few weeks, which potentially exerted anti-inflammatory effects and induced a transient improvement in cholestasis. However, although concomitant corticosteroid therapy may have contributed to early biochemical improvement, the durable radiologic response and consistency with published reports support T-DXd activity in this setting. Regarding survival outcomes, a case comparable to ours was reported by Luo S et al., describing a patient with HER2-positive mBC who experienced early progression after adjuvant dual anti-HER2 blockade and developed pulmonary lymphangitic carcinomatosis; treatment with T-DXd achieved a median PFS of 14.2 months (28). Similarly, we observed for our patient, with an aggressive disease course with early progression (<12 months) to first-line therapy, a PFS of around 12 months (29). Notably, available evidence suggests that VC carries may hold an independent negative prognostic value (30). Indeed, the aggressive and extensive presentation, by contrast, for example, with oligometastatic disease, likely reflects a distinct biology background associated with treatment resistance and poorer outcomes. Therefore, prospective, ideally head-to-head studies are urgently needed to give insights into this unexplored setting and to advance beyond chemotherapy-based strategies in favor of novel, more effective agents (21, 31–34). From this point of view, international guidelines are beginning to reflect this paradigm shift, for example by recognizing ADCs (T-DXd, sacituzumab govitecan, datopotamab deruxtecan) and poly (ADP-ribose) polymerase inhibitors (olaparib, talazoparib) as potential alternatives to conventional chemotherapy in luminal mBC with VC (35, 36), although their integration into clinical practice remains challenging. Barriers include not only the limited evidence on efficacy, safety, and dosing, but also considerations of cost, toxicity (e.g., ILD risk in pulmonary VC), as well as patient condition and preference. Moreover, access and reimbursement often require prior therapy for advanced disease, progression on (neo)adjuvant regimens, beyond of course the confirmation of the molecular target. Therefore, regular pathological and/or liquid biopsy assessment and re-evaluation throughout the disease course remains essential to avoid missing potential opportunities for novel therapies (37). Importantly, the ABC guidelines explicitly acknowledge that, when an ADC cannot be promptly accessed, HER2-positive patients with severe organ dysfunction may be treated with weekly paclitaxel plus trastuzumab and pertuzumab, or with oral capecitabine plus anti-HER2 therapy, as alternative cytotoxic backbones (4).

**Table 1:** Evidence of T-DXd use in patients with VC.

Study reference [authors, year] and type	Patient characteristics [age, sex, biologicalsubtype]	Number of prior metastatic treatment lines	Type and presentation of VC	Initial T-DXd dose (mg/kg)	Clinical response	Biochemical response	Best radiological response	Survival outcome	T-DXd dose escalation	Toxicity related to T-DXd
Higashiyama N. 2020 (24) Case report	45y; Female; Luminal B/HER2+ mBC	4	Hepatic Failure Total Bilirubin: 6.6 mg/dL; ALP 1188 U/L; AST 250 U/L; ALT 42 U/L	4 mg/kg (25% dose-reduced)	PS ECOG improvement 3→1 and pain relief (at C1D17)	Total bilirubin level improvement (at C1D17) and normalization (at C3)	Extracranial and intracranial response (at C2D6)	Ongoing response (at C9)	4 → 5.4 mg/kg (at C3)	G1 infusion reaction (at C1) G3 anemia (after C1)
Sun L. 2024 (25) Case series	1. 53y; Female; Luminal B/HER2+ mBC 2. 49y; Female; Luminal A/HER2-low mBC; 3. 53y; Female; Luminal B/HER2-low mBC	1. 3 2. 3 3. 3	Hepatic Failure 1. Total Bilirubin 10.4 mg/dL; AST 49 U/L; ALT 19 U/L; ALP 87 U/L 2. Total Bilirubin 12 mg/dL; ALP 876 U/L; ALT 285 U/L; AST 1636 U/L 3. Total Bilirubin 9.6 mg/dL	1. 4.4 mg/kg 2. 3.2 mg/kg 3. 3.2 mg/kg	1. Stability, mobility, mental clarity improvement (at C2) 2. NR 3. Clinical improvement (appetite, weight, activity) (NR the cycle)	1. Total bilirubin decrease (after C1) 2. Liver function tests improvement (at C2) and total normalization (at C4) 3. Total bilirubin reduction (at C2) and normalization (at C4)	1. NR 2. Liver response (at C4) 3. Liver response (after C4)	1. PFS 4 months 2. PFS 8 months 3. NR	1. Maintained 4.4 mg/kg 2. 3.2→ 4 (at C3) → 5.4 mg/kg (at C4) 3. Maintained 3.2 mg/kg	1. G4 urinary infection and fever and hepatic encephalopathy (after C1). -G4 Fever and hyperbilirubinemia (after C2) 2. Not specified (“excellent tolerance”) 3. “Mild expected side effects” (not further specified) (at C2)
Damaj N. 2024 (26) Case report	56y; Female; HER2-low mBC	3	Hepatic Failure Total Bilirubin: 7.1 mg/dL; AST 659 U/L; ALT 145 U/L; GGT 1254 U/L; ALP 812 U/L; albumin 32 g/L; INR 1.32	3.6 mg/kg	Clinical improvement (consciousness, decreased ascites/jaundice) (after C1)	Liver function tests improvement (at C1D15) and total normalization (after C2)	Liver PR (at C2)*	NA	NR	NR
Kapoor N (27). Case report	52y; Female; Luminal/ HER2-low mBC	2	Hepatic Failure Total Bilirubin: 19.0 mg/dL; AST 1449 U/L; ALT 666 U/L; ALP 640 U/L; albumin 37 g/L; INR 1.32	4.4 mg/kg	Gastro -intestinal symptoms and overall functional capacity improvement (at C5)	- AST/ALT and Total bilirubin levels reduction (at C2) - AST/ALT levels normalization (at C3) - Total bilirubin level normalization (at C5)	SD (at C5)	PFS: 18 months	4,4 → 3.2 mg/kg (from C2 maintained till C25)	Poor oral intake, diarrhea, atrial flutter, myelosuppression requiring transfusion support (at C1) From C2 well tolerated
Luo S (28). Case report	59y; Female; HER2+ mBC	0	PLC Radiological appearance and clinical symptoms	NR	PS ECOG along with chest tightness, dyspnea, cough, and headaches markedly improved	NA	Extracranial and intracranial PR* (NR the cycle)	PFS: 14.2 months	NR	G1 ILD (NR the cycle)

The table summarizes published real-world evidence describing the use of T-DXd in patients with mBC presenting with VC. Reported data include baseline clinical characteristics, diagnostic findings, therapeutic management, details of T-DXd administration, survival outcomes, and safety profile. .

*The reported reduction in disease parameters allows for the definition of a radiological response criterion according to RECIST v1.1 (for intracranial disease RANO-BM criteria), or as directly stated by the authorsTrastuzumab deruxtecan (T-DXd), Cycle Number (C) and Day (D) of T-DXd administration, Metastatic breast cancer (mBC),Visceral crisis (VC), Human epidermal growth factor receptor 2-positive (HER2+), pulmonary lymphangitic carcinomatosis (PLC), interstitial lung disease (ILD), Aspartate Aminotransferase (AST), Alanine Aminotransferase (ALT), Gamma-Glutamyl Transferase (GGT), Alkaline Phosphatase (ALP), Serum Albumin (Albumin), International Normalized Ratio (INR), progression-free survival (PFS), Partial response (PR), Stable disease (SD), Progressive disease (PD), Not reported (NR), Not applicable (NA), Milligrams per kilogram (mg/kg), Response Evaluation Criteria In Solid Tumors, version 1.1. (RECIST 1.1), Response Assessment in Neuro-Oncology – Brain Metastases (RANO-BM).

Moreover, future investigations should incorporate a consensual and uniform definition of VC, particularly for less clearly defined entities beyond hepatic and pulmonary involvement (e.g., bone marrow, peritoneal, brain, or meningeal disease), to ensure comparability of patient cohorts and generate reliable evidence. For example, the applicability of the positive findings from the phase II randomized RIGHT-CHOICE trial to daily practice is indeed limited by its broad and subjective definition of VC for enrollment (38). Lastly, while patients with moderate liver dysfunction and hepatic metastases were eligible in pivotal T-DXd trials without mandatory dose adjustments (7, 8, 10, 20, 23, 39, 40), safety data and dosing guidance in the setting of severe hepatic dysfunction (whether disease-related or not) remains also in this case lacking. This is particularly relevant since T-DXd carries an inherent risk of hepatotoxicity and is primarily metabolized by hepatic CYP450 enzymes, raising the risk of further liver function deterioration or severe “rebound” extrahepatic toxicities, including major cardiac and pulmonary events (17). However, along with reassuring real-world evidence, showing a lower incidence of hepatotoxicity than in clinical trials (~5% vs 25-35% any grade and 0–0.5% grade ≥3 vs 2–4%) (10, 11), our case similarly to all the other reports, displayed that low-dose T-DXd was well tolerated and appeared safe in case of severe hepatic impairment. Furthermore, despite some heterogeneity, a similar cautious dose-escalation approach of T-DXd as liver function improved, was consistently adopted across them and, in the absence of formal guidelines, appears to be a reasonable, safe, and effective strategy. To conclude, our case adds to the limited evidence supporting T-DXd as a safe and effective option for HER2-positive mBC complicated by disease-related hepatic VC, with stepwise dose-escalation as a reasonable strategy in this setting. More broadly, aggressive BC subtypes such as triple negative and HER2-positive are typically characterized by rapid disease progression and a higher incidence of liver metastases, and indications for T-DXd continue to expand the HER2 expression spectrum (39, 41). Collectively, these trends increase the likelihood of encountering in routine clinical practice patients with significant disease-related hepatic dysfunction who may be candidates for T-DXd.

Further evidence is therefore needed to ensure that this unprecedented therapeutic option is not inappropriately withheld when indicated as standard of care, or even preferentially considered in the setting such as VC.

## 4 Patient perspective

From the patient’s perspective, the initiation of T-DXd represented both a moment of uncertainty and hope. She described the rapid deterioration of her liver function as “the most frightening phase” of her illness, marked by the fear of running out of effective options. An additional source of concern was the return to intravenous treatment, which she associated with a symbolic step into a more advanced and serious phase of her illness. Starting treatment during such instability was emotionally challenging, yet she reported feeling reassured by the close monitoring and clear communication from the care team. Within the first weeks, the clinical improvement, mirroring the biochemical recovery, was perceived as “a turning point,” giving her renewed confidence in the therapy. She described the subsequent months on T-DXd as a period in which she could “resume a sense of normality,” with manageable side effects and a preserved daily life. Even at the time of progression, she expressed gratitude for the meaningful period of disease control and the possibility to transition to another treatment while still feeling clinically well.

## Data Availability

The datasets presented in this article are not readily available because the data presented in this study consist of individual clinical information (e.g., laboratory results, radiologic imaging) derived from the patient’s medical records. Due to patient confidentiality and privacy considerations, these data are not publicly available. Further information may be provided by the corresponding author upon reasonable request and in compliance with ethical standards. Requests to access the datasets should be directed to giuseppe.saltalamacchia@humanitas.it.
